# How can we facilitate adoption and scale for early detection of Alzheimer’s disease? Insights from implementing a community‐based screening program using cognitive assessments and RetiSpec screening in Ontario, Canada

**DOI:** 10.1002/alz.086147

**Published:** 2025-01-09

**Authors:** Sharon Cohen, Jennifer Giordano, Alissa Kurzman, Amelia Hansen, Michelle Martinez, Negar Sohbati, Naeem Abdulla, Colette Cameron, Shmuel Estreicher, Sangeeta Semwel, Eliav Shaked, Catherine C Bornbaum

**Affiliations:** ^1^ Toronto Memory Program, Toronto, ON Canada; ^2^ RetiSpec, Toronto, ON Canada; ^3^ University of California Irvine, Irvine, CA USA; ^4^ Davos Alzheimer’s Collaborative, Philadelphia, PA USA; ^5^ Victoria Village Optometry, North York, ON Canada; ^6^ Summerhill Optometry, Toronto, ON Canada; ^7^ Alzheimer Society of Toronto, Toronto, ON Canada

## Abstract

**Background:**

A critical need to increase Alzheimer’s disease (AD) screening exists given rising incidence, new disease‐modifying treatments, and ill‐equipped primary care settings. This study assessed the feasibility of a novel, community‐based AD screening program to increase cognitive and retinal‐based assessments.

**Methods:**

An observational study, supported by the Davos Alzheimer’s Collaborative, assessed the utility of leveraging community‐based settings to increase rates of cognitive assessment (conducted by Alzheimer Society (AS) social workers [SWs]) and RetiSpec’s AI‐based eye test in optometry settings to detect biologic signatures of AD (plus participant survey). Eligible participants were adults aged ≥55 years with self‐reported memory concerns. Primary and secondary endpoints were to increase cognitive assessment rates and have 10% of assessments originate from optometry, respectively. A utilization‐focused evaluation explored risks, benefits, facilitators, and barriers to these settings from frontline providers’ perspectives.

**Results:**

N = 916 individuals were screened (60.2% from optometry) with N = 134 participants enrolled (age: 74.3 years±7.0; 63.7% female; 39.5% minority groups). N = 124 participants received cognitive assessments: 118 Montreal Cognitive Assessment (mean score: 24.7±3.7), 124 Boston Naming Test (13.3±2.0), 6 Mini‐Mental State Exam (18.7±5.7) and 6 Clock Drawing Test (1.2±1.2). N = 120 participants discussed their results with a clinician (37.9% with the NP). N = 25 dementia specialist referrals were made, N = 52 received an AD‐related diagnosis, along with medication prescription (N = 5), further medical investigation (N = 30) and clinical monitoring (N = 27). N = 99 underwent a RetiSpec scan (N = 65 predicted positive). Surveys (N = 71) demonstrated a positive scan experience (4.2±1.3/5) and unanimous interest in sharing results with a PCP (5.0±0.0/5). Endpoint 1 was met with 13.8 assessments/month completed versus 1.8/month in the pre‐study period. Endpoint 2 was met with 29.0% of assessments originating from optometry.

Evaluation findings indicate that screening programs should consider: promoting brain health awareness in community settings; utilizing clinicians with AD training for screening and follow‐up activities; leveraging optometry clinics and AS’s to enable broad access to screening; and adopting technology that leverages existing infrastructure (i.e., RetiSpec’s eye test) to enable scalable increases to AD screening.

**Conclusions:**

Utilizing community‐based settings, including optometry clinics for RetiSpec screening and AS SWs for cognitive screening, led to meaningful improvements in early detection and subsequent medical care.

